# Comparative Proteomic Analysis of Sweet Orange Petiole Provides Insights Into the Development of Huanglongbing Symptoms

**DOI:** 10.3389/fpls.2021.656997

**Published:** 2021-04-19

**Authors:** Bo Li, Yi Zhang, Dewen Qiu, Frédéric Francis, Shuangchao Wang

**Affiliations:** ^1^The State Key Laboratory for Biology of Plant Diseases and Insect Pests, Institute of Plant Protection, Chinese Academy of Agricultural Sciences, Beijing, China; ^2^Functional and Evolutionary Entomology, Gembloux Agro-Bio Tech, University of Liège, Gembloux, Belgium

**Keywords:** citrus greening, huanglongbing, *Candidatus* Liberibacter, proteomics, defense response, tradeoff, leaf petiole

## Abstract

Huanglongbing (HLB) is the most destructive citrus disease worldwide. This is associated with the phloem-limited bacterium *Candidatus* Liberibacter, and the typical symptom is leaf blotchy mottle. To better understand the biological processes involved in the establishment of HLB disease symptoms, the comparative proteomic analysis was performed to reveal the global protein accumulation profiles in leaf petiole, where there are massive HLB pathogens of *Ca.* L. asiaticus-infected Newhall sweet orange (*Citrus sinensis*) plants at the asymptomatic and symptomatic stages compared to their healthy counterpart. Photosynthesis, especially the pathway involved in the photosystem I and II light reactions, was shown to be suppressed throughout the whole *Ca.* L. asiaticus infection cycle. Also, starch biosynthesis was induced after the symptom-free prodromal period. Many defense-associated proteins were more extensively regulated in the petiole with the symptoms than the ones from healthy plants. The change of salicylic and jasmonic acid levels in different disease stages had a positive correlation with the abundance of phytohormone biosynthesis-related proteins. Moreover, the protein–protein interaction network analysis indicated that an F-type ATPase and an alpha-1,4 glucan phosphorylase were the core nodes in the interactions of differentially accumulated proteins. Our study indicated that the infected citrus plants probably activated the non-unified and lagging enhancement of defense responses against *Ca.* L. asiaticus at the expense of photosynthesis and contribute to find out the key *Ca.* L. asiaticus-responsive genes for tolerance and resistance breeding.

## Introduction

Huanglongbing (HLB) or “citrus greening” is a destructive and incurable citrus disease that dramatically impairs citrus industries worldwide (Graça et al., 2016; Munir et al., 2018). HLB disease is first reported in Asia and is widely distributed in more than 40 countries and regions in Asia, Oceania, Africa, and the Americas (Munir et al., 2018). It is associated with a gram-negative and phloem-inhabiting bacterium of the genus *Candidatus* Liberibacter, including three known species, “*Ca.* L. asiaticus,” “*Ca.* L. americanus,” and “*Ca.* L. africanus” (Wang et al., 2017). It is known that *Ca.* L. asiaticus is the dominant pathogenic of the three species (Miles et al., 2017). It is frustrating that all known citrus varieties and their relatives can be infected with the *Ca.* L. asiaticus pathogens. The pathogens are normally transmitted by grafting with HLB-infected budwoods and by phloem-feeding psyllid, *Diaphorina citri* and *Trioza erytreae* (Lopes et al., 2009). HLB-infected citrus plants usually develop a variety of symptoms, such as vein yellowing and hardening, and blotchy mottling and chlorosis that are sometimes mistaken for element deficiency. At the advanced symptomatic stage, the massive *Ca.* L. asiaticus resides and propagates in the phloem of citrus trees that causes phloem blockage and aberrations, excessive starch accumulation, callose depositions, and disruption of grana structures in chloroplast (Graça et al., 2016). Consequently, the fruits of infected trees are small, lopsided, and premature, with a bitter, acidic flavor as well as aborted seeds (Gottwald et al., 2007). Desperately, once the pathogen settles on citrus trees, the pathogen spreads to all tissues quickly and leads to plant death after a period of several months to a few years (Killiny et al., 2018).

Traditional management practices such as citrus psyllid control and infected tree eradication have little success in controlling this disease. Breeding of HLB-resistant varieties also failed to achieve satisfactory results due to the lack of specific candidate resistance genes (Wang, 2019). Therefore, identifying key genes and proteins of citrus plants in response to *Ca.* L. asiaticus infection is essential not only to better understand the mechanisms behind plant–microbe interactions but also to supply potential candidate genes that enhance citrus tolerance (or even resistance) to HLB for further genetic engineering breeding and to control disease development.

Several studies have been conducted through “omic” methods, including transcriptomics, proteomics, and metabolomics, to investigate the changes of genes, proteins, and metabolites induced by *Ca.* L. asiaticus infection in leaf (Killiny and Nehela, 2017a, b; Nehela et al., 2018; Nehela and Killiny, 2019, 2020), root (Zhong et al., 2015; Padhi et al., 2019), stem (Aritua et al., 2013), fruit (Yao et al., 2019, 2020), and calyx abscission tissue (Zhao et al., 2019). Because of the uneven distribution of *Ca.* L. asiaticus in the infected plants, *Ca.* L. asiaticus could induce similar or distinct responses between different tissue materials. For example, transcriptional analyses indicated that the overall expression pattern in *Ca.* L. asiaticus-infected stems was highly similar to infected leaves. *Ca.* L. asiaticus infection can induce the accumulation of starch, sucrose, and glucose and affect other diverse cellular functions, such as the phloem blockage and aberrations, cell wall modification, activation of plant defense, and hormone biosynthesis (Aritua et al., 2013). Another transcriptomic analysis of leaves and fruits of Valencia orange showed the disorder and opposite expression of sucrose and starch metabolism in fruits and leaves of naturally infected trees, which caused the disruption in the source and sink tissue relationship (Martinelli et al., 2013). These reports indicated that different tissue types are likely to hold tissue-specific responses during *Ca.* L. asiaticus infection. Acquisition of nutrients from residence is essential for the colonization and proliferation of *Ca.* L. asiaticus populations during different disease stages. Also, as *Ca.* L. asiaticus is an obligate pathogen in the citrus phloem sieve tube, the interactions with *Ca.* L. asiaticus in the phloem tissues represent the more accurate and comprehensive responses of citrus plants to pathogen infection. The petiole is the transition between the stem and the leaf blade that contains vascular tissue, providing a connection from the stem to permit sap to enter the leaf and serving to transport the photo-assimilate from leaf to other parts of the plant. There is limited knowledge to date about the proteomic differences in leaf petioles, where the *Ca.* L. asiaticus pathogen accumulate and reproduce more than leaves and other tissues, to unravel the response of the citrus plant to the threat of HLB disease.

The aim of this work was to perform a proteomic approach based on tandem mass tag (TMT) labeling to characterize the comparative changes in the leaf petiole proteome of *Ca.* L. asiaticus-infected citrus plants in asymptomatic and symptomatic stages. Further protein–protein interaction (PPI) analysis with differentially accumulated proteins identified from *Ca.* L. asiaticus-infected petioles compared to their healthy counterpart revealed specific node proteins that may play an important role in HLB disease symptom development. Together with the comparative assay of physiological indexes between HLB-infected and healthy citrus plants, such as chlorophyll and plant hormone content. Our findings highlight molecular mechanism and physiological processes related to the development and formation of typical HLB symptoms and provide candidate targets for HLB controlling and resistance breeding through a more complete understanding and mining of *Ca.* L. asiaticus-responsive proteins in petioles.

## Materials and Methods

### Plant Material

Two-year-old sweet orange (*Citrus sinensis* cv. Newhall) trees grafted on citrange (*C. sinensis* [L.] Osb × *Poncirus trifoliata* [L.] Raf.) rootstock were selected as plant materials in this study. Twenty prepared seedlings were graft-inoculated with bud sticks from *Ca.* L. asiaticus-infected and healthy citrus trees, respectively. The inoculated trees were detected and verified by quantitative PCR (qPCR) with *Ca.* L. asiaticus-specific primers (Li et al., 2006). After inoculation, all plants were maintained under controlled conditions at 25–28°C, 70 ± 5% relative humidity, and a natural photoperiod in an insect-proof greenhouse. *Citrus* plants were irrigated once per week and fertilized every 6 weeks using 12-6-5 NPK fertilizer. Samples were collected from *Ca.* L. asiaticus-inoculated and mock-inoculated citrus trees at 16 and 22 weeks after grafting (WAG), respectively. CI1 represents the asymptomatic sample collected from *Ca.* L. asiaticus-infected citrus trees at 16 WAG, and CI2 represents the symptomatic sample collected from *Ca.* L. asiaticus-infected citrus trees at 22 WAG. While MI1 represented the sample collected from mock-inoculated citrus trees at 16 WAG, MI2 represented the sample collected from mock-inoculated citrus trees at 22 WAG. In all cases, four fully expanded leaves per tree were taken from the topmost leaves of different branches located at the same level of ramification. Each biological replicate included a pool of eight leaves. Three biological replicate samples of HLB-infected and healthy plants were collected to extract total proteins for proteomic analysis. Because work on *Ca.* L. asiaticus pathogen is subject to governmental regulations in China, the authors employed biosafety protocols to manage potential risks and prevent dissemination of the pathogen: (i) Operations of plant samples were carried out in a separate biosafety cabinet, and all experimental consumables that may contact with *Ca.* L. asiaticus pathogen during the experiment were autoclaved; (ii) *Ca.* L. asiaticus-inoculated plants were restricted to independent greenhouses; and (iii) all *Ca.* L. asiaticus-inoculated plants were destroyed by autoclaving when the experiment was completed.

### Quantification of *Ca.* L. asiaticus Genomes

*Ca.* L. asiaticus population in grafting inoculated citrus plants was obtained by qPCR using an absolute quantification protocol. Starting at 12 WAG, each plant was tested every 4 weeks; the method refers to a previous study (Li et al., 2006). Briefly, petiole and blade samples were ground separately to powder in liquid nitrogen. Total DNA from the 200-mg fresh tissues was extracted using a DNeasy Plant Mini Kit (Qiagen, Beijing, China). Then, 1 μl of a 100 μl DNA elution was used for quantitative analysis of the *Ca.* L. asiaticus population. The standard qPCR reaction condition was 95°C for 30 s followed by 40 cycles at 95°C for 5 s and 60°C for 34 s. All amplification was performed on ABI7500 Real-Time PCR system with SYBR Fast qPCR Mix (Takara, Dalian, China). A standard curve was generated by performing serial dilutions of recombinant plasmid containing a fragment of 16S rRNA from *Ca.* L. asiaticus. The mean Ct values were then entered into the standard equation for calculating *Ca.* L. asiaticus genome equivalents in the sample: *Y* = –0.2753*X* + 10.68 (*R*^2^ = 0.9973), where *Y* is the estimated log concentration of plasmid templates and *X* is the Ct values of qPCR.

### Plant Protein Extraction, Digestion, and TMT Labeling

Leaf petiole tissues were ground into powder in liquid nitrogen and were precipitated according to the manufacturer’s protocol using a ProteoExtract Protein Precipitation kit (CalBiochem, Darmstadt, Germany). Two hundred micrograms of protein sample was diluted by buffer (100 mM Tris, pH 8.0, 8 M urea) to 100 μl and then 11 μl of DTT (1 M) was added to the solution, which was incubated at 37°C for 1 h. The treated samples were added into a 10-kDa ultrafiltration tube (Millipore, MA, United States) and centrifuged at 12,000 *g* for 10 min. Then, 100 μl of 55 mM iodoacetamide (IAA) was added to the ultrafiltration tube and incubated for 20 min protected from light at room temperature. Subsequently, 50 mM triethylammonium bicarbonate (TEAB) was used as exchange buffer. Then, proteins were tryptic digested with sequence-grade modified trypsin (Promega, WI, United States) overnight at 37°C in a 1:50 trypsin-to-protein mass ratio, and the desalted and lyophilized peptide mixture was labeled using chemicals from the TMT reagent kit (Pierce Biotechnology, IL, United States). Samples were then desalted and dried by vacuum centrifugation.

### HPLC Fractionation

The trypsin-digested peptides were fractionated by high pH reverse-phase HPLC using XBridge C18 column, 5 μm particles, 4.6 mm ID, and 250 mm length (Waters Corporation, MA, United States) on an Ultimate 3000 system (Thermo Fisher Scientific, MA, United States). Briefly, peptides were first separated with a gradient of 5% to 45% buffer B (20 mM ammonium formate in 80% ACN, pH 10.0) over 40 min. The column temperature was maintained at 30° and flow rate was maintained at 1 ml/min. Then, the peptides were combined into 12 fractions and dried by vacuum centrifuging.

### LC-MS/MS Analysis

Mass spectrometric data were collected on an Orbitrap Fusion Tribrid mass spectrometer (Thermo Scientific, MA, United States) coupled to a an Easy-nLC 1000 system (Thermo Scientific, MA, United States). The tryptic peptides were dissolved in 0.1% formic acid, and 10 μl of peptide sample was loaded onto a 2-cm Acclaim C18 Pepmap trap column, with a flow rate of 10 μl/min for 3 min and subsequently separated on a 75 μm × 15 cm Acclaim Pepmap RSLC analytical column (Thermo Scientific, MA, United States) using a 120 min gradient at a flow rate of 300 nl/min with buffer D (ACN with 0.1% formic acid) ranging from 3% to 32%. The electrospray voltage of 2 kV versus the inlet of the mass spectrometer was used. The fusion mass spectrometer was set at data-dependent mode, allowing the apparatus to automatically switch between MS and MS/MS acquisition. Survey full-scan MS spectra (m/z 350–1550) were acquired in Orbitrap at a resolution of 120K followed by MS/MS using high-energy collisional dissociation (HCD) at a resolution of 30 K. The AGC target was set to 4 × 10^5^ and the precursor isolation window was 1.6 m/z. MS/MS fixed first mass at 110 m/z. One microscan was recorded using 45-s dynamic exclusion in all cases. The mass spectrometry proteomics data have been deposited to the ProteomeXchange Consortium via the iProX partner repository with the dataset identifier PXD023576.

### Database Searches and Bioinformatics Analysis

Tandem mass spectra were extracted and charge state was deconvoluted using Mascot Distiller version 2.6.1. All MS/MS samples were analyzed using Mascot, which was set up to search the UniProt_*Citrus sinensis* database assuming the digestion enzyme trypsin. The Mascot program was conducted with a fragment ion mass tolerance, 0.020 Da, and a parent ion tolerance, 10 ppm. The modifications due to TMT6plex of lysine and the n-terminus and carbamidomethyl of cysteine were specified in Mascot as fixed modifications. Oxidation of methionine and deamidation of asparagine and glutamine and acetyl of the n-terminus were specified in Mascot as variable modifications. The MS/MS-based peptide and protein identifications were validated using Scaffold (version 4.6.2). According to the Scaffold Local FDR algorithm, peptide identifications were accepted if they could achieve an FDR of less than 1.0%. The acceptance of protein identification was based on an FDR of less than 5% and contains at least two unique peptides. Proteins that contained similar peptides and cannot be distinguished based on MS/MS analysis alone were classified to satisfy the principles of parsimony. To quantitate TMT label-based quantitation peptide and protein identifications, we used Scaffold Q + in this study. Across samples, normalization was performed iteratively on intensities as previously described (Oberg et al., 2008). Subsequently, spectra data were log-transformed, pruned of those matched to multiple proteins, and weighted by an adaptive intensity weighting algorithm. Differentially accumulated proteins (DAPs) were determined using a Mann-Whitney Test with a significance level set at *p* < 0.05 corrected by the Benjamini-Hochberg method.

### Enrichment Analyses

Enriched gene ontology (GO) term annotations of the upregulated and downregulated DAPs were identified using Singular Enrichment Analysis (SEA) in agriGO v.2 (Tian et al., 2017), which is a bioinformatics platform specially focus on agricultural species. The hypergeometric test with a Yekutieli (FDR under dependency) correction was chosen to identify the significant GO terms (*p* < 0.05). The Kyoto Encyclopedia of Genes and Genomes (KEGG) enrichment analysis was performed by using ClueGO (Bindea et al., 2009) that is embedded in Cytoscape software. Significant pathways were identified using a two-sided hypergeometric test (*p* < 0.05).

### Functional Categorization

To better display the pathway enrichment result, MapMan and its embedded PageMan software were used (Schwacke et al., 2019). The fold change values (FC) of the whole set of DAPs were subjected to the MapMan software. Based on the pathways database of Citrus Sinensis downloaded mappings, the functionalities of DAPs were analyzed using MapMan. Meanwhile, a statistics-based and visualized overview of changed pathways were provided by using PageMan application with the Wilcoxon rank-sum test. This method pinpoints which gene functional subcategories were upregulated and downregulated in each pairwise comparison.

### PPI Network Analysis

The DAPs were analyzed using the STRING online server^1^ and obtained PPI data were used to construct the network maps in CytoScape software version 3.7.2 with an interaction score > 0.7 (high confidence). In the networks, the nodes correspond to the proteins and the edges represent the interactions. To detect highly connected regions of the network, ClusterONE 1.0 was used with the following basic parameters (minimum size = 5, minimum density = 0.05, and edge weights = combined score) (Nepusz et al., 2012). Results for ClusterONE indicated that predicted clusters tended to consist of proteins in the same cellular component, and these proteins are likely to have similar functions and/or participate in the same biological process.

### Detection of Chlorophyll Content

HLB-infected and healthy leaves in the asymptomatic and symptomatic stage were separately collected. Five hundred milligrams of leaf tissue was ground and then incubated in 50 ml of 80% acetone at 4° in the dark. After 2 h, the absorbance of chlorophyll extraction was tested with a UV-visible spectrophotometer at 645- and 663-nm wavelength separately, and the chlorophyll content was calculated according to the previously reported formulas (Porra et al., 1989). Each treatment was repeated six times.

### Determination of the Endogenous Levels of Salicylic Acid and Jasmonic Acid

For the salicylic acid (SA) and jasmonic acid (JA) content assays, HLB-infected and healthy citrus samples in the asymptomatic and symptomatic stage were collected in three replicates. For each sample, 1 g of the leaf petiole tissue was extracted and quantitated for free SA and JA, as describedy previously with minor modifications (Matsuura et al., 2009). Briefly, the petiole tissue was ground and then soaked in 10 ml of methanol-H_2_O-acetic acid (80:19:1, v:v). After extraction overnight at 4°C, the supernatant was collected by centrifuging at 13,000 rpm for 15 min at 4°C. The pellet was re-extracted with the aforementioned solution and all supernatants were pooled. The mixture was dried in a speed vacuum with heat (∼40°) and was subjected to solid-phase extraction using Sep-Pak C18 cartridges and lyophilized. The sample was resuspended in 0.5 ml of 80% methanol with 1% acetic acid, filtered, and analyzed by Ultra Performance Liquid Chromatography (UPLC). It was performed on a Waters ACQUITY ethylene-bridged (BEH) C18 column (1.7 μm, 50 mm × 2.1 mm) at 40° with a flow rate of 0.4 ml/min. The analytes were eluted from the column with a mixed buffer A (water with 0.1% acetic acid) and buffer B (methanol with 0.1% acetic acid) using a linear gradient mode as follows: 10% B (90% A) for initial 3 min, 10% B (90% A) to 90% B (10% A) for 3–4 min, and isocratic 10% B was finally maintained from 4 to 7 min. The authenticity of the SA/JA from citrus petiole extract was verified based on the retention times and spectral properties, which matched perfectly to those of commercial SA/JA standards.

### Gene Expression Validation

To evaluate the reliability of identified DAPs in proteomics analysis, 16 proteins were selected based on their functions and fold change of the proteomics result, and their relative transcription levels were analyzed by qRT-PCR. Briefly, total RNA was isolated from citrus petioles using an RNeasy Plant Mini kit (Qiagen, Valencia, CA). RNA reverse transcription was conducted using the PrimeScript RT Reagent Kit with gDNA Eraser (Takara, Dalian, China). Gene expression was measured using SYBR Fast qPCR Mix (Takara, Dalian, China) on an ABI7500 Real-Time PCR system. *Citrus* glyceraldehyde-3-phosphate dehydrogenase (*GADPH*) gene was used as a housekeeping gene to normalize the amount of RNA in each sample. The specific primers used for RT-qPCR were designed using NCBI Primer-BLAST^2^, which are listed in Supplementary Table 6. The relative mRNA expression levels were calculated from the threshold cycle using the ΔΔCt method (Schmittgen and Livak, 2008). Each experiment was performed in triplicate.

### Preparation of Polyclonal Antibody and Western Blot Analysis

The purified proteins psaDb (CISIN_1g028581mg) and GBSS1 (CISIN_1g007224mg) were used as antigens for antibody production. Antibodies were produced in rabbit by Huaan Biotechnology Company (Hangzhou, China). For Western blot analysis, the total protein of *Citrus* petioles was extracted and the implementation method refers to *Section 2.3*. Total proteins (20 μg) from each sample were resolved on 12% SDS-PAGE, and the protein was transferred onto a polyvinylidene fluoride membrane (GE Healthcare, Chicago, Illinois, United States). The blots were analyzed using respective primary antibodies at a dilution of 1:500 in PBS. Protein bands were visualized using BeyoECL Star kit (Beyotime, Shanghai, China).

## Results

### Determination of *Ca.* L. asiaticus Titer in Leaf Midribs and Blade

**FIGURE 1:**
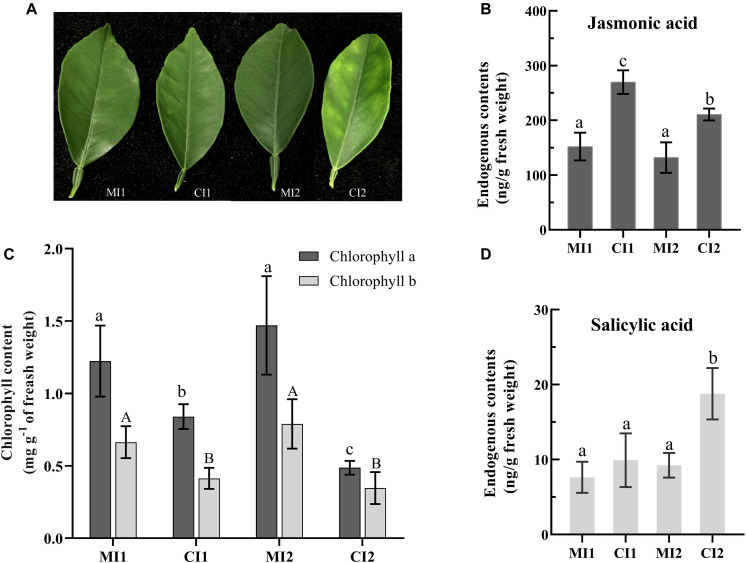
**(A)** The leave types used in the experiment. The level of jasmonic acid **(B)** and salicylic acid **(D)** in leaf petioles. **(C)** Content of chlorophyll in citrus leaf tissues. Representative leaves and their chlorophyll and phytohormone contents of MI1, CI1, MI2, and CI2. Different letters indicate significant differences at *P* < 0.05, determined using one-way ANOVA with Tukey’s *post hoc* test. Error bars represent the SD. MI1 and MI2 represent the sample collected from mock-inoculated citrus trees at 16 and 22 weeks after grafting, respectively. CI1 and CI2 represent asymptomatic sample collected at 16 weeks after grafting and symptomatic sample collected at 22 weeks after grafting from *Ca.* L. asiaticus-inoculated citrus trees, respectively.

Huanglongbing-infected leaves initially appeared to have representative HLB symptoms of asymmetrical blotchy mottling from about 20 WAG (Figure 1A). *Ca.* L. asiaticus titer in petiole and blade tissues were separately measured at multiple time points. Samples from *Ca.* L. asiaticus-inoculated citrus trees (CI1 and CI2) were checked to be infected with *Ca.* L. asiaticus, while the samples from mock-inoculated trees (MI1 and MI2) were found to be free of *Ca.* L. asiaticus (Table 1). Moreover, the *Ca.* L. asiaticus titer in petiole tissues was significantly higher than that of blade tissues, especially in the samples collected at 16 and 20 WAG, the difference was more than 80 times. Therefore, selecting petiole tissues in the early stages of HLB disease could truly and accurately represent the changes of the symptom formation in response to *Ca.* L. asiaticus infection. According to the results of *Ca.* L. asiaticus detection and the appearance time of visual symptoms in HLB-infected citrus trees, samples were collected from *Ca.* L. asiaticus-inoculated and corresponding healthy *Citrus* plants at 16 and 22 WAG for further proteomics analysis, respectively.

**TABLE 1:** Detection and quantification of *Ca.* L. asiaticus genomes by quantitative polymerase chain reaction (qPCR) in petiole and blade tissues of HLB-infected citrus plants.

	Ct value		*Ca.* L. asiaticus population (gene copies/g of fresh tissues)	
		
Sampling time (WAG)	Petiole	Blade	Petiole	Blade
12	32.18	38.75	3.32 × 10^4^	4.32 × 10^2^
16	28.68	35.18	3.04 × 10^5^	4.95 × 10^3^
20	22.81	29.68	1.26 × 10^7^	1.61 × 10^5^
24	21.09	26.67	3.75 × 10^7^	1.09 × 10^6^

*The standard equation Y = –0.2753X + 10.68 (R^2^ = 0.9973) was used to convert individual Ct values into bacterial population as genome equivalents per gram of fresh tissues. Data represent the mean of triplicate experiments. Ct, crossing threshold; WAG, weeks after grafting.*

### Overview of Protein Level Accumulation Changes in Response to *Ca.* L. asiaticus Infection

All MS/MS samples were analyzed using Mascot (Matrix Science, London, United Kingdom; version 2.5.1) and searched against the UniProt_*Citrus sinensis* database. Firstly, a large number of peptides were identified based on the MS data (Supplementary Table 1). The length of most peptides distributed was between 6 and 14 amino acids, which agrees with the property of tryptic peptides (Supplementary Figure 1). The distribution of peptide delta mass in each replicate was between –1.2 and 2.2 ppm, and the number of spectra for each protein in different replicates showed consistency, which means the mass accuracy of the MS data fit the requirement. Meanwhile, those proteins with a cutoff value of > | ± 1.3| -fold and a *p* value < 0.05 were considered as DAPs. Since the goal of the study was to determine the response of *Citrus* plants to HLB infection, two sets of comparisons were developed, namely, CI1 vs. MI1 and CI2 vs. MI2. There were 786 (607 upregulated and 179 downregulated) and 385 (214 up regulated and 171 downregulated) DAPs for CI1 vs. MI1 and CI2 vs. MI2, respectively (Supplementary Table 2).

### Enrichment Analyses of DAPs for the Significant Biological Processes and Pathways

**FIGURE 2:**
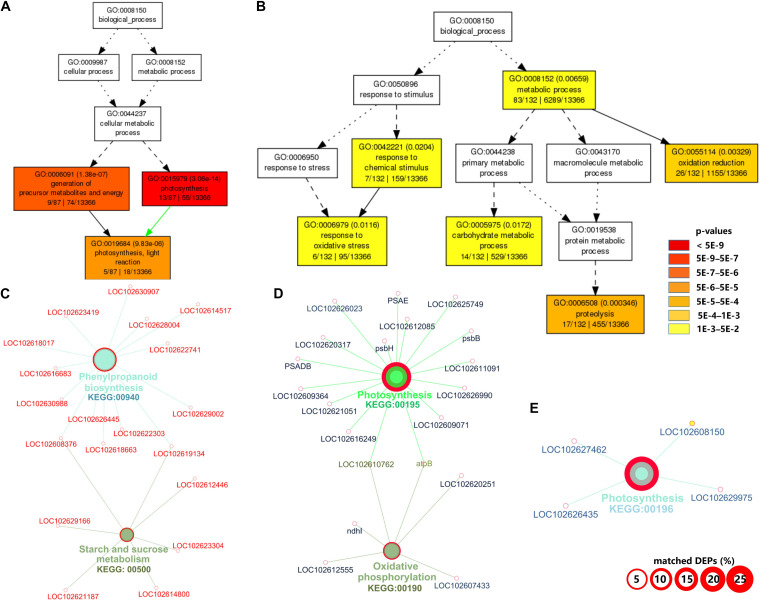
Gene ontology (GO) and Kyoto Encyclopedia of Genes and Genomes (KEGG) pathway enrichment analyses of differentially accumulated proteins (DAPs) between CI1 and MI1. Significant GO terms under the category of “Biological Process” for downregulated **(A)** and upregulated **(B)** proteins. Statistical test method: hypergeometric, and multi-test adjustment method: Yekutieli (FDR under dependency). The significant (term *p* value < 0.01) KEGG pathways related to downregulated **(C)** and upregulated proteins **(D,E)**. The width of the red border represents the proportion of matched DAPs in the corresponding KEGG term (%).

The complete list of GO terms and KEGG pathways are provided in Supplementary Table 3. For downregulated DAPs in CI1, the significantly enriched GO terms in biological process were those related to photosynthesis (GO:0015979), generation of precursor metabolites and energy (GO:0006091), and photosynthesis and light reaction (GO:0019684) (Figure 2A). The downregulated pathways in CI1 included photosynthesis (KEGG:00195 and KEGG:00196), oxidative phosphorylation (KEGG:00190), glutathione metabolism (KEGG:00480), and protein processing in endoplasmic reticulum (KEGG:04141) (Figures 2D,E). The significantly upregulated biological process for CI1 included defense response (GO:0006979 and GO:0042221), metabolic process (GO:0008152), carbohydrate metabolic process (GO:0005975), oxidation reduction (GO:0055114), and proteolysis (GO:0006508) (Figure 2B), while the upregulated pathways in CI1 were the phenylpropanoid biosynthesis (KEGG:00940), glyoxylate and dicarboxylate metabolism (KEGG:00630), and starch and sucrose metabolism (KEGG:00500) associated with starch cleavage (Figure 2C and Supplementary Table 3).

**FIGURE 3:**
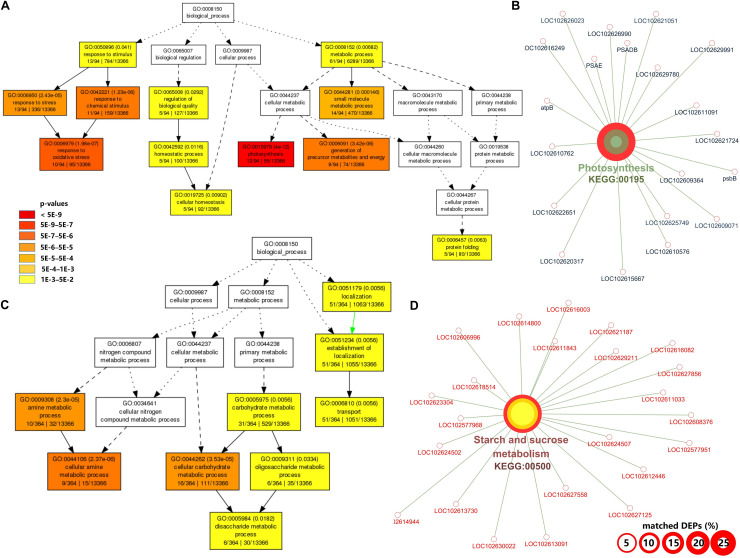
Gene ontology (GO) and Kyoto Encyclopedia of Genes and Genomes (KEGG) pathway enrichment analysis of differentially accumulated proteins (DAPs) between CI2 and MI2. **(A,B)** Significant GO terms under the category of “Biological Process” for upregulated **(A)** and downregulated **(B)** proteins. Statistical test method: hypergeometric, and multi-test adjustment method: Yekutieli (FDR under dependency). **(C,D)** The significant (term *p* value < 0.01) KEGG pathways related to upregulated **(C)** and downregulated genes **(D)**. The width of the red border represents the proportion of matched DAPs in the corresponding KEGG term (%).

At the symptomatic stage in CI2 (Figure 3), the significantly upregulated biological process GO terms were related to transport (GO:0006810, GO:0051234, and GO:0051179), carbohydrate metabolic process (GO:0005975), cellular carbohydrate metabolic process (GO:0044262), oligosaccharide metabolic process (GO:0009311), disaccharide metabolic process (GO:0005984), and amine metabolic process (GO:0009308 and GO:0044106) (Figure 3B). The significantly upregulated pathway in CI2 mainly included starch and sucrose metabolism (KEGG:00500) and N-Glycan biosynthesis (KEGG:00510) (Figure 3D and Supplementary Table 3). The most significant biological process in downregulated DAPs was involved in photosynthesis (GO:0015979); the remaining biological processes were related to defense response (GO:0050896, GO:0006950, GO:0042221, and GO:0006979), protein folding (GO:0006457), regulation of biological quality (GO:0065008), homeostatic process (GO:0042592 and GO:0019725), and generation of precursor metabolites and energy (GO:0006091) (Figure 3A), while the downregulated pathways in CI2 were involved in photosynthesis (KEGG:00195), carbon fixation in photosynthetic organisms (KEGG:00710), glyoxylate and dicarboxylate metabolism (KEGG:00630), and protein processing in endoplasmic reticulum (KEGG:04141) (Figure 3C and Supplementary Table 3).

Among the biological processes and pathways, photosynthesis was the most downregulated biological process at both asymptomatic and symptomatic stages. However, the GO enrichment analysis indicated that the biological process associated with the response to oxidative stress and the oxidation reduction was upregulated in CI1 (Figure 2B), while they were downregulated in CI2 (Figure 3A). Meanwhile, the upregulated amine metabolic process and the downregulated cellular homeostasis were unique biological processes in CI2 compared with CI1 (Figures 2, 3).

### Functional Categorization of DAPs

Graphical format illustrating differentially regulated functional groups was provided (Supplementary Figure 2). Inspection of the display revealed several general trends in both sets of samples (CI1 vs. MI1 and CI2 vs. MI2): *Ca.* L. asiaticus infection downregulated photosynthesis (PS) involved in photosystem II light reaction and abiotic stress-related proteins. Meanwhile, the distinctive repressed functional groups in CI2 were photosynthetic electron carrier ferredoxin, ATP synthase, Calvin cycle, thioredoxin redox pathway, and ascorbate and glutathione redox pathway. Instead, no unique pathway was significantly downregulated in CI1 vs. MI1. Moreover, MapMan software was used to annotate and visualize the functional classes, which had significant differences at the asymptomatic and symptomatic stages (Supplementary Table 4). The analyses showed that DAPs from CI1 and CI2 were grouped into diverse cellular functional categories mainly including photosynthesis, carbohydrate metabolism, cell and cell wall metabolism, secondary metabolism, hormone metabolism, lipid metabolism, amino acid and protein degradation, transport processes, and misc (*O*-methyl transferases, cytochrome P450, GDSL-motif lipase). These corresponded to potential key proteins related to the establishment of HLB disease symptoms in asymptomatic and symptomatic *Citrus* trees.

### Photosynthesis Process

**FIGURE 4:**
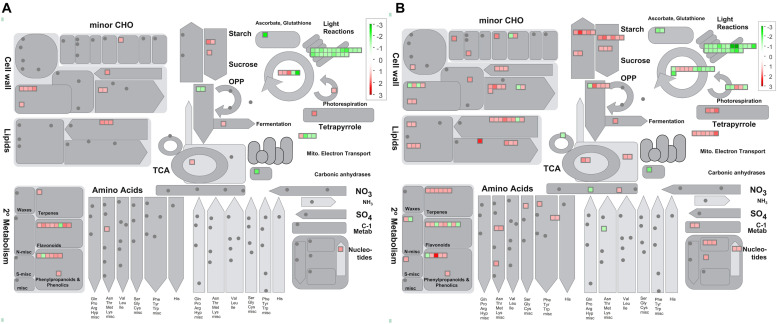
Overview of metabolic pathways that are regulated by *Ca.* L. asiaticus infection in leaf petioles. **(A)** Asymptomatic stage (CI1 vs. MI1) and **(B)** symptomatic stage (CI2 vs. MI2). Red squares, significantly upregulated proteins; green squares, significantly upregulated proteins. Each colored square represents a single annotated gene in a particular pathway.

Abundance of dozens of proteins in CI1 and CI2 involved in light reactions of photosynthesis decreased significantly with infection by *Ca.* L. asiaticus, including several light-harvesting chlorophyll a/b binding proteins, PSII polypeptide subunits, PSI polypeptide subunits, ATP synthase, and electron carrier ferredoxin (Figure 4 and Supplementary Figure 2). The normal accumulation of these proteins is a prerequisite for photosynthesis, which ensures the operation of a series of processes of capturing light energy, electron transport, and conversion into chemical energy in chloroplast thylakoid membrane. Also, most proteins involved in photorespiration and Calvin cycle processes in CI2 were repressed compared with CI1; the details of DAPs related to photosynthesis process are listed in Supplementary Table 4.

Besides, the accumulation of chlorophyllase (cisin_ 1g020188mg) was significantly increased in both asymptomatic and symptomatic stages (Supplementary Table 4). This illustrated the possibility of direct disruption of chlorophyll synthesis or disintegrating the chloroplast in HLB-infected *Citrus* trees by regulating the accumulation of chlorophyllase. Meanwhile, the chlorophyll concentration of *Citrus* leaves from asymptomatic to HLB symptoms showed significant statistical differences as shown in Figure 1C. Although the chlorophyll content of HLB-infected *Citrus* trees in CI1 and CI2 decreased significantly compared with the corresponding healthy trees, the chlorophyll content in asymptomatic *Citrus* trees was still higher than the ones with visible symptoms (Figure 1C).

### Carbohydrate Metabolism

At the asymptomatic stage, one beta-fructofuranosidase insoluble isoenzyme and two amylases involved in starch and sucrose degradation were upregulated (Table 2 and Supplementary Figure 3). No significant change in starch synthesis pathway was observed in CI1 compared with healthy *Citrus* trees. At the symptomatic stage, the response of *Citrus* trees on *Ca.* L. asiaticus infection was very contradictory in carbohydrate metabolism. Besides the three abovementioned proteins that were also upregulated in CI21, six other over-accumulated proteins involved in sucrose and starch degradation were identified in CI2 (Table 2 and Supplementary Figure 3), including two sucrose synthases, a phosphoglucan water dikinase, a phosphoglucan phosphatase, an alpha-amylase, and an alpha-glucan phosphorylase. Also, seven proteins related to sucrose and starch synthesis in CI2 were significantly induced (Table 2 and Supplementary Figure 3), including two sucrose-phosphate synthases, two starch synthase, and two 1,4-alpha-glucan branching enzyme (GBE) and a Glucose-1-phosphate adenylyltransferase (glgC).

**TABLE 2:** Differently accumulated proteins involved in the sucrose and starch metabolism in response to *Ca.* L. asiaticus infection.

Gene Name UniProt	Description	Pathway	CI1 vs. MI1^a^		CI2 vs. MI2^b^	
			Fold change	*p* value	Fold change	*p* value
CISIN_1g007857mg	Beta-fructofuranosidase insoluble isoenzyme	Sucrose degradation	1.41	5.29E-02	1.34	1.87E-02
CISIN_1g003661mg	Sucrose synthase				1.41	4.49E-02
CISIN_1g036539mg	Sucrose synthase 5				1.32	3.56E-02
CISIN_1g010067mg	Beta-amylase	Starch degradation	1.32	6.53E-03	1.32	2.18E-02
CISIN_1g014447mg	Alpha-amylase		1.75	5.39E-03	1.63	1.89E-03
CISIN_1g009825mg	Phosphoglucan water dikinase				1.35	4.87E-02
CISIN_1g040474mg	Phosphoglucan phosphatase DSP4				1.47	1.27E-02
CISIN_1g002585mg	Alpha-amylase 3				1.90	3.41E-02
CISIN_1g003196mg	Alpha-glucan phosphorylase 2				1.74	8.45E-03
CISIN_1g014638mg	Sucrose-phosphatase 2	Sucrose synthesis			1.48	2.97E-03
CISIN_1g001705mg	Probable sucrose-phosphate synthase 4				1.34	9.48E-03
glgC	Glucose-1-phosphate adenylyltransferase large subunit	Starch synthesis			1.53	1.80E-02
CISIN_1g002609mg	1,4-alpha-glucan-branching enzyme 3				1.36	1.62E-01
CISIN_1g035501mg	1,4-alpha-glucan-branching enzyme 1				1.72	9.28E-03
CISIN_1g006091mg	Starch synthase 1				1.47	7.39E-03
CISIN_1g007224mg	Granule-bound starch synthase 1 (GBSS1)				2.16	1.01E-02

*^a^: CI1 vs. MI1 mean asymptomatic petiole and the healthy counterpart; ^b^: CI2 vs. MI2 mean asymptomatic petiole and the healthy counterpart.*

### Cell Wall Organization

The plant cell wall is a resilient and stable barrier composed of complex polysaccharides, which plays a critical role in providing structural support and host defense against pathogens. There were 8 and 18 proteins found related to cell wall metabolism among the DAPs in CI1 and CI2, respectively (Figure 6 and Supplementary Table 4). The DAPs involved in cellulose degradation, an endoglucanase with cellulase activity and a beta-glucosidase, were upregulated in CI1 (Figure 6), and those two proteins were also upregulated in CI2. Besides, three other DAPs, namely, a cellulase 3 (cel3), a beta-glucosidase, and an endoglucanase, were only upregulated in CI2. In this work, the proteins involved in pectin demethylesterification were differently regulated between CI1 and CI2 (Supplementary Table 4). It should be noted that all of them were enhanced in CI1, including three pectin methylesterases, whereas no significant difference was observed in CI2. Conversely, two other pectin methylesterases were repressed in CI2. For CI2, it was found that the abundance of polygalacturonase family protein increased and the abundance of polygalacturonase inhibiting protein 1 (PGIP1) decreased. The different accumulation patterns of these two enzymes at the symptomatic stage may synergistically exacerbate the degradation of pectin components of the plant cell wall.

**FIGURE 5:**
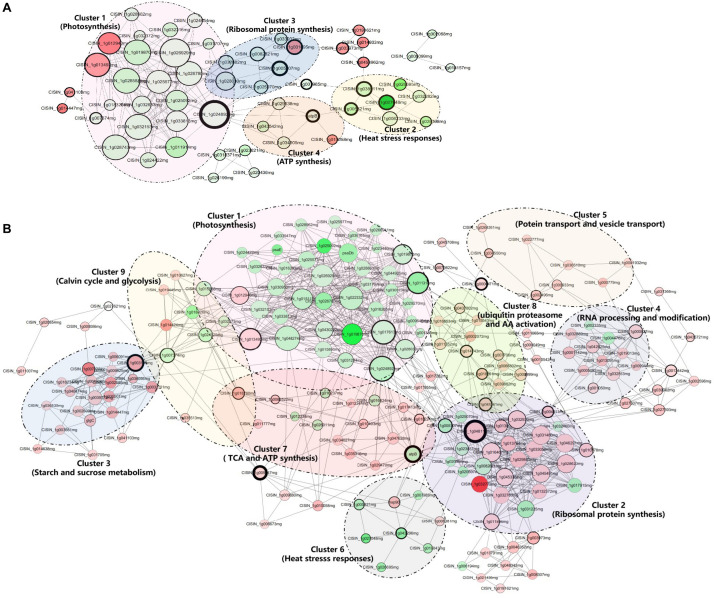
Interaction network of differentially accumulated proteins (DAPs) analyzed by Cytoscape software at the asymptomatic **(A)** and symptomatic **(B)** stage. Each cluster is a set of highly connected nodes detected by ClusterONE. The size of the nodes and the width of the black border of nodes are positively correlated to the Degree Centrality and Betweenness Centrality. The upregulated and downregulated proteins in the clusters were shown in red and green, respectively, and the color correlates to the fold change of differentially accumulated proteins. Detailed information on node and a protein list are provided in Supplementary Table 5.

**FIGURE 6:**
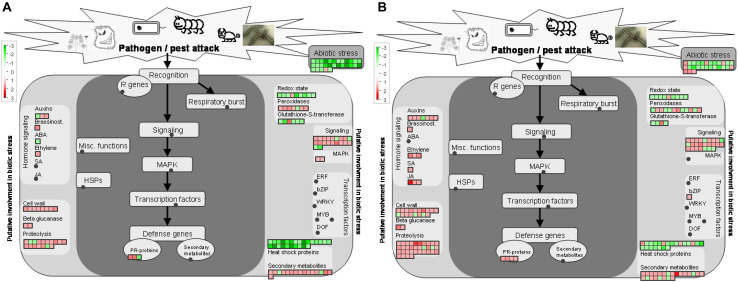
Regulation of proteins involved in stress responses protein by *Ca.* L. asiaticus infection in leaf petioles. **(A)** Asymptomatic stage (CI1 vs. MI1) and **(B)** symptomatic stage (CI2 vs. MI2). Red squares, significantly upregulated proteins. Each colored square represents a single annotated gene in a particular pathway. No significantly upregulated proteins involved in ubiquitin-dependent protein degradation were observed in the asymptomatic stage.

### Secondary Metabolism Pathway

Secondary metabolism plays critical roles in helping the plants to reinforce plant resistance when subjected to adverse environmental conditions. In this work, the abundance of proteins involved in biosynthesis of isoprenoids and phenylpropanoids was mostly upregulated in both CI1 and CI2 (Figure 4 and Supplementary Table 4). In addition, more proteins related to isoprenoid metabolism were induced in CI2 than in CI1 (Supplementary Table 4). Of eight upregulated proteins related to flavonoids metabolism in *Ca.* L. asiaticus-infected *Citrus* trees, one chalcone synthase 3 (CHS3) was upregulated in CI1 and downregulated in CI2. CHS3 is a key enzyme involved in the biosynthesis of flavonoids. Four *O*-methyl transferases that are involved in the synthesis of a variety of secondary metabolites including phenylpropanoids and alkaloids were upregulated in CI2 but showed no significant difference in CI1.

### Hormone Metabolism

Plants are adapted at adjusting phytohormones to cope with biotic and abiotic stresses. There were 6 and 15 DAPs related to hormone metabolism in CI1 and CI2, respectively (Figure 6 and Supplementary Table 4). Among them, a down-accumulated auxin-responsive family protein and several up-accumulated auxin-induced proteins were identified in both CI1 and CI2. Furthermore, three DAPs involved in JA metabolism were upregulated in CI2. Two of those proteins were lipoxygenases (cisin_1g002649mg, cisin_1g002839mg), which are key enzymes involved in JA synthesis. Notably, a S-adenosyl-L-methionine-dependent methyltransferase (cisin_1g045960mg), which is plausibly related to SA synthesis, was induced in CI2. However, those proteins associated with JA and SA biosynthesis were not upregulated at the asymptomatic stage.

To further determine the impact of *Ca.* L. asiaticus infection on the hormone metabolic pathways in *Citrus* trees. The content of JA and SA in HLB-infected and healthy leaf petioles at the asymptomatic and symptomatic stages were measured, respectively. The JA content in HLB-infected leaf petioles was significantly increased compared with their healthy counterpart in both disease stages (Figure 1B). SA content in symptom samples was significantly increased in response to *Ca.* L. asiaticus infection, whereas no significant difference was found in the asymptomatic stage (Figure 1D).

### Stress and Defense Responses

There were 16 and 17 proteins related to biotic stress responses to HLB infection in identified DAPs at the asymptomatic and symptomatic stages, respectively (Figure 6 and Supplementary Table 4). Among these proteins, eight had the same regulation mode, and two downregulated proteins included stress-related proteins, one endochitinase, and six upregulated proteins including two transaminase transferase activity proteins, an NBS-LRR class disease resistance protein, a thaumatin-like protein, and two dirigent-like proteins. The analysis of the remaining DAPs in CI1 showed that all nine proteins were upregulated (Figure 6A), mainly including one stress-enhanced protein, two chitinase-like proteins, a PR-4 like protein with chitin binding activity, and a trypsin and protease inhibitor family protein that was conversely regulated in CI2, while most of the remaining proteins were downregulated in CI2 except one thaumatin-like protein belonging to the PR-5 family that was upregulated (Figure 6B). The downregulated DAPs mainly included two NBS-LRR class disease resistance proteins, three thaumatin-like proteins, and one chitinase-like protein. At the same time, most heat shock proteins were downregulated at two disease stages.

In addition, six of the seven peroxidases in DAPs were induced at the asymptomatic stage in response to HLB infection (Supplementary Table 4), while the differential abundance of 11 peroxidases was detected at the symptomatic stage compared with corresponding healthy *Citrus* trees. Of those peroxidases, six were downregulated and five were upregulated (Supplementary Table 4).

### Amino Acids and Protein Metabolism

In general, amino acids and protein metabolism were affected by abiotic and biotic stress. In this study, only one S-adenosylmethionine synthase involved in amino acid biosynthesis was upregulated in the asymptomatic stage (Supplementary Table 4). This situation changed completely at the symptomatic stage; eight upregulated proteins mainly associated with central amino acid biosynthesis, aspartate family biosynthesis, and tryptophan biosynthesis were upregulated (Supplementary Table 4). Furthermore, DAPs involved in ubiquitin-dependent protein degradation pathway were only identified at the symptomatic stage (Supplementary Figure 4). There were 12 ubiquitin-related DAPs that were found in symptomatic *Citrus* trees compared with their healthy counterpart. Among them, five ring-type E3 ubiquitin ligases, an F-box family protein, a BTB/POZ and MATH domain-containing protein, and three 26S proteasome subunits were significantly enhanced. This result indicated that ubiquitin-mediated protein degradation may be initiated from the symptomatic stage in response to HLB infection.

### PPI Network Construction and Analysis of DAPs

The PPI networks on DAPs of CI1 vs. MI1 (Figure 5A) and CI2 vs. MI2 (Figure 5B) were built and used to identity 4 and 11 clusters of highly interconnected nodes in pre-symptomatic and symptomatic stages, respectively. The associated functions displayed on the PPI networks and detailed information are listed in Supplementary Table 5. All the protein complexes obtained from the PPI network analysis of the CI1 vs. MI1 group can be found in the CI2 vs. MI2 group. The enriched clusters associated with photosynthesis and abiotic stress responses presented similar downregulated protein accumulation patterns in two groups. The opposite accumulation patterns in both groups are mainly involved in ribosomal protein and ATP synthesis. In addition, several clusters related to primary metabolic processes with unregulated accumulation patterns, such as carbohydrate metabolism and amino acid and protein metabolism, were extra identified in CI2 vs. MI2 group. No significant cluster related to plant hormones and defenses was found from PPI networks data in two groups.

In the PPI network of the DAPs related to the CI1 vs. MI1 group, proteins such as CISIN_1g024892mg (downregulated; ATP synthase subunit delta) and ATP B (downregulated; ATP synthase CF1 beta subunit) had higher degree centrality and betweenness centrality, which indicated that it was likely to have more interactions and connectivity with other proteins as key nodes (Figure 5A). In the CI2 vs. MI2 group (Figure 5B), the above two proteins also have relatively high degree centrality and betweenness centrality. In addition, CISIN_1g048118mg (upregulated; guanine nucleotide-binding protein) and CISIN_1g003196mg (upregulated; Alpha-1,4 glucan phosphorylase) with high degree of connectivity were only found in the CI2 vs. MI2 group.

### Quantitative RT-PCR and Western Blot Validation

In order to validate the proteomics data by qRT-PCR, 16 proteins involved in different biological processes were selected, which were significantly modulated in HLB disease progression. The related transcript of these proteins includes photosynthesis, carbohydrate metabolism, stress and defense response pathways, and hormone metabolism (Supplementary Figure 5A). Additionally, the squared correlation coefficient values between the fold change from qRT-PCR and proteomic experiments in CI1 vs. MI1 and CI2 vs. MI2 were 0.6057 and 0.8907, respectively (Supplementary Figure 5B). These data indicated that the quality of proteomic analysis was reliable. Also, the accumulation levels of GBSS1 (granule-bound starch synthase 1) and psaDb (PS1 reaction center subunit III) were analyzed using Western blot. The accumulation of CsGBSS-1 in the HLB-infected *Citrus* plant was higher than the healthy counterpart at the symptomatic stage but showed no significant difference at the asymptomatic stage to the healthy counterpart. At both stages, the accumulation trend of CsPSAE-1 was decreased in response to *Ca.* L. asiaticus infection. The Western blotting results confirmed the reliability and accuracy of the proteomic data in this study (Supplementary Figure 6).

## Discussion

HLB disease is a highly destructive and obligate pathogenic *Citrus* disease that is seriously harmful to the global *Citrus* industry. Once *Ca.* L. asiaticus bacteria successfully invaded the phloem, disease control was found to be very difficult to apply to cure infected host plants. Although pathogens can be found in most tissues of HLB-affected *Citrus* trees, the leaf midrib and petiole were usually used to detect the presence of *Ca.* L. asiaticus because the pathogen exceeded other *Citrus* tissues (Li et al., 2009). Our results were consistent with this statement. In addition, the *Ca.* L. asiaticus titer increased sharply during the transition from pre-symptomatic (16 WAG) to symptomatic (20 WAG) stage, which was probably caused by the rapid propagation of pathogens after successful colonization in petiole of HLB-infected trees. The petiole, as a stalk that twist the leaf to face the sun, was responsible for the two-way transportation of photosynthetic products and nutrients between source and sink organs. The anatomical analyses presented that the ultrastructural damages of phloem cells from HLB-affected petiole tissues includes the deposition of callose plugs throughout the phloem elements, and the swelling of sieve elements and companion cell walls, which is believed to hinder the transport of photo-assimilates (Etxeberria and Narciso, 2015). Therefore, a comparative proteomic approach was used in the present study to comprehensively update the knowledge of protein accumulation profiles of petiole tissues in response to *Ca.* L. asiaticus infection and to provide deeper insights into the HLB symptomology that were discussed below.

### Photosynthesis Components as Primary Target of *Ca.* L. asiaticus Infection

Since chloroplasts master a number of defense-related pathways, it was not surprising that pathogen may actively disrupt chloroplastic functions, presumably to inhibit chloroplast-derived defense signals (Kangasjärvi et al., 2012). Previous omics studies revealed that *Ca.* L. asiaticus infection could generally repress photosynthetic pathway in infected tissues of HLB-susceptible *Citrus* varieties, and lower levels of associated proteins were identified as early as 8 weeks post-grafting compared to HLB-free plants (Fan et al., 2012; Martinelli et al., 2013; Ramsey et al., 2020). Although symptomatic fruits (small and green) over-expressed genes involved in photosynthesis compared to asymptomatic and apparently healthy fruits (ripe and yellow), the opposite expression pattern in the comparison between asymptomatic and apparently healthy fruit from HLB-infected trees and healthy fruit from HLB-free trees and the accumulation of several chlorophyll a-b binding protein were downregulated and chlorophyllase was induced (Martinelli et al., 2012, 2013). These findings showed that the photosynthetic system in the HLB-infected fruits was also sabotaged to a substantial degree at the early disease stage. Concurrently, our results also verified that photosynthesis components such as the downregulation of light-harvesting complex in photosystem I and II, electron transport, and ATP synthesis were decreased and the upregulation of chlorophyllase probably was the reason for detecting a reduction of chlorophyll levels in HLB-affected *Citrus* plants in this study. The most characteristic symptom of HLB disease, leaf yellowing with blotchy mottle, was likely to be due to chlorophyll degradation in infected plants. *Ca.* L. asiaticus infection altered the total citrus leaf photosynthetic pigment balance and promoted chlorophyll degradation, which contributed to the development of symptoms (Killiny and Nehela, 2017b). We found our results of the colorimetric-determined chlorophyll content to be consistent with this study. Meanwhile, the citrus response system was simulated by significantly induced ABA concentration in citrus plants with *Ca.* L. asiaticus infection (Killiny and Nehela, 2017b). Based on the evidence above, we posed a speculative hypothesis that the *Ca.* L. asiaticus-infected *Citrus* plants showed insufficient and lagging enhancement of defense responses against *Ca.* L. asiaticus at the expense of photosynthesis. Also, the inhibition of photosynthesis in tolerant varieties was less affected by *Ca.* L. asiaticus infection (Hu et al., 2017; Deng et al., 2019), showing that ability to maintain photosynthesis during infection was a vital element of defense. Taking our study and the published data together, this strongly suggested that photosynthesis progresses were proactively modulated throughout the whole *Ca.* L. asiaticus infection cycle, and the intensity of destruction varied according to different tissues and varieties.

### Deregulation of Starch Biosynthesis as a Hallmark of HLB Symptom Establishment

HLB-mediated massive accumulation of starch in the chloroplast and sieve elements was usually found at the advanced disease stage. Physiological analysis showed that the leaves with symptoms were statistically superior in concentration of starch in comparison with HLB-free leaves (Etxeberria et al., 2009; Manzanilla-Ramírez et al., 2019). Moreover, anatomical analyses revealed that the starch accumulation can be distinctly seen in the petiole of tolerant rough lemon and susceptible sweet orange symptomatic leaves. In the HLB-affected but symptomless leaves, the starch granules were hardly observed (Fan et al., 2012). In addition, gene expression profile of tolerant Mexican lime and susceptible Washington navel orange in response to *Ca.* L. asiaticus infection uncovered that the expression of multiple genes related to starch synthesis was significantly induced at the symptomatic stage, while no difference at the asymptomatic stage compared to healthy counterparts was observed (Arce-Leal et al., 2020; Chin et al., 2020). The consistent results from this study at the protein level confirmed the increased abundance of starch synthesis-related proteins such as glgC, GBSS1, and GBE in the petiole of leaves with typical symptoms. We speculated that the disorder of starch biosynthesis was due to direct enhanced starch synthesis, which began in the transition period from the asymptomatic to the symptomatic stage. Also, the backlog of excessive starch in sieve tubes impeded the export of photoassimilates from source leaves, which further stimulated redundant sugar conversion into starch in the chloroplasts until this organelle structure was disrupted (Etxeberria et al., 2009). Ultimately, such disturbances of starch metabolism and the upregulation of chlorophyllase played a synergistic effect to cause chlorosis and mottle symptoms by disintegrating the chloroplast in later stages of disease progression.

### Growth–Defense Trade-Offs of HLB-Infected *Citrus* Plants

Plant–microbe interactions are usually invisible but constantly occur in many different ways and at many different levels. Virtually, plants have to adapt and resist to pathogen invasions by adjusting their growth and activating defense responses, which is often referred to growth–defense trade-offs (Neuser et al., 2019). However, initiating a defensive response is accompanied by the cost of reduced growth and reproduction (Karasov et al., 2017; Neuser et al., 2019). Significant differences in the protein accumulation profiles upon *Ca.* L. asiaticus infection were observed from the asymptomatic to symptomatic stage of *Citrus* petioles, which possibly helped to illustrate the underlying molecular mechanism for growth–defense trade-offs in response to *Ca.* L. asiaticus.

Implementation of immune reactions and biosynthesis of protective compounds put a huge demand for energy and resources. Then, the negative impact of resources reallocation was manifested in diminishing of photosynthesis, which generates ATP, NADPH, and carbohydrates (Huot et al., 2014). Such metabolic shift was paradoxical and required host plants to achieve a balance between growth and defense to optimize plant fitness. Obviously, sweet oranges had not made a good trade-off in response to *Ca.* L. asiaticus infection. Normally, the inhibition of plant growth–defense trade-off on photosynthesis was limited to infected cells, which can be offset by increased photosynthesis in surrounding cells, and the stability of most photosynthetic proteins accepted a temporary stop at the transcriptional level without impacts on photosynthesis itself (Huot et al., 2014). In our study, the abundance of photosynthetic proteins was decreased continuously after *Ca.* L. asiaticus infection. Also, chlorophyll degradation occurred in all types of infected leaves meaning difficulty to have enough source leaves to compensate for the loss of photosynthesis. In addition, chloroplasts are major generators of reactive oxygen species (ROS) that is central to plant response to several pathogens in early stages of infection (Kangasjärvi et al., 2012). A previous study found that *AtCLH1* (Chlorophyllase 1 of *Arabidopsis thaliana*) RNAi silenced plants displayed greatly increased resistance to *Erwinia carotovora* infection resulting from increased ROS levels. Combined with our results, it was speculated that the upregulation of chlorophyllase in infected *Citrus* plants probably resulted in increased susceptibility to *Ca.* L. asiaticus, accompanied by the reduction of photosynthesis efficiency. However, whether the decline in photosynthesis of infected *Citrus* plants is a programmed part of the Growth–defense trade-offs or simply a by-product of pathogen induction remains to be determined.

The hormonal crosstalk seems to play a considerable role in regulating the growth–defense trade-off. There is mounting evidence to support the notion whereby resources normally allocated toward growth are diverted to support hormone signaling-mediated defense (Huot et al., 2014; Züst and Agrawal, 2017). Many phytohormones, such as auxin, ethylene, AS, and JA, can specifically manipulate host immune reactions and susceptibility to pathogen infection (Karasov et al., 2017). In the present study, both hormones accumulated to high levels at the later stage of *Ca.* L. asiaticus infection. This result may seem paradoxical because SA and JA are usually antagonistic hormones implicated in the immune responses against biotroph and necrotrophy organisms, respectively. Based on the current literature, this seems to be explained by the non-canonical mechanism that JA signaling pathway following SA accumulation was activated through the SA receptors, instead of the canonical JA receptor, to promote plant immunity (Liu et al., 2016). However, metabolic costs involved in the sustained expression of defenses are ubiquitous and can be considerable. The long activation of SA and JA can inhibit plant growth by modifying the auxin signaling (Huot et al., 2014). We conjectured that phytohormone-regulated responses of infected *Citrus* plants to *Ca.* L. asiaticus infection lags behind the development of HLB disease, resulting in the inability of the *Citrus* plant to respond appropriately and timely to HLB pathogens in the early stages of infection. Failure of growth–defense trade-off in infected tissues that the costs of defense ultimately impacted plant fitness. From another point of view, it would be worth studying whether SA and/or JA treatments could modify the time courses of HLB disease appearance. Application of phytohormone suppressed the progression of HLB disease severity. Hu et al. (2018) found that the trunk injections of SA reduced *Ca.* L. asiaticus titer by 45.5% to 65.8% and provided 30.0% to 36.6% disease control in treated citrus trees as compared with water-injected control trees. Similar results were obtained through exogenous application of SA to control HLB in the field (Li et al., 2017). Moreover, similar studies showed that pre-exposure of citrus plants to H_2_O_2_ or SNP stimulus can prime an enhanced plant defense against biotic or abiotic stress (Tanou et al., 2009). It is becoming increasingly evident that priming techniques (e.g., external application of natural or synthetic compounds in plants) can improve the tolerance of crops to environmental challenges (Tanou et al., 2012). Therefore, it is reasonable to assume that the pre-treatment of SA significantly reduced disease severity and slowed down HLB disease progress.

Each defensive trait or compound requires precursor molecules and energy from primary metabolism of plant for synthesis, modification, transport, and maintenance or storage (Züst and Agrawal, 2017). Upon *Ca.* L. asiaticus infection in susceptible *Citrus* plants, we found that the enhanced metabolic changes are involved in carbohydrate, amino acid, and protein metabolism. A similar phenomenon at the transcriptional level was also observed in previous studies (Martinelli et al., 2016; Hu et al., 2017). These findings are likely to support the notion that citrus hosts persistently supply the precursor substances for the synthesis of hormones and secondary metabolites by adjusting the primary metabolism at the site of infection. Nonetheless, visual symptoms of HLB disease in infected leaves still appeared after a certain incubation period, by which time the production of plant protection products may already be ineffective. Regrettably to our knowledge, it has not been found that the effective and specific bioactive end products were accumulated at the right times and places, and in amounts sufficient to fulfill their functions to control *Ca.* L. asiaticus. Such enhanced metabolic activity at high cost not brought the efficient contribution of secondary metabolites and hormones in infected *Citrus* to immunity requires in response to *Ca.* L. asiaticus, we even speculated that such metabolic regulation was manipulated by HLB pathogens.

Plants and microbes interact constantly to modulate their growth and development. Obviously, *Ca.* L. asiaticus seemed to be a winner in pathogen–most *Citrus* species interactions. Resources acquisition from phloem is crucial for the successful establishment of *Ca.* L. asiaticus. Recent studies found that *Ca.* L. asiaticus pathogens competed with hosts to directly scramble ATP or utilize glucose and other carbohydrates from colonized phloem tissues for supporting colonization and proliferation (Jain et al., 2017; Cruz-Munoz et al., 2018). Whether it was manipulated by *Ca.* L. asiaticus or constrained by growth–defense trade-off of host, preventing sucrose and starch export from infected cells cannot be viewed as mere coincidence. In addition, *Ca.* L. asiaticus pathogens have undergone reductive genome evolution, lacking genes related to lipopolysaccharide biosynthesis and various secretion systems that are commonly associated with the pathogen-triggered recognition and activation of plant immunity (Wulff et al., 2014). This may be an important factor to avoid stimulating plant defense reaction. Likewise, the study by Li et al. (2017) reported that the citrus HLB pathogen *Ca.* L. asiaticus can encode a functional SA hydroxylase to degrade SA and suppress plant defenses. Correspondingly, there were few changes in accumulation of defensive and hormone signaling-related proteins at the early stages of infection in our study. The above results showed that *Ca.* L. asiaticus escaped recognition of the *Citrus* immune system and successful colonization leads to the postponement of defense response that should be activated at the initial infection phase and site, and the subsequent deregulation of multifold physiological processes may be synergistically affected by pathogen attack and self-adjustment between growth and defenses. Meanwhile, our PPI network analyses indicated that the most important node of significant cluster associated with photosynthesis is an F-type ATPase, whereas alpha-1,4 glucan phosphorylase is the core node of starch and sucrose metabolism in *Citrus-Ca.* L. asiaticus interactions. Therefore, we speculated that these two proteins may be important mediated proteins of the growth–defense trade-off in response to *Ca.* L. asiaticus infection, which provided candidate regulatory switches to optimize the allocation of resources for improving *Citrus* tolerance or resistance.

## Conclusion

In conclusion, the present study revealed a global protein accumulation profile in *Ca.* L. asiaticus-infected *Citrus* petiole at different disease stages. The photosynthetic system in the *Citrus* petiole was sabotaged to a substantial degree by *Ca.* L. asiaticus from the early disease stage, accompanied by a continuous decline in chlorophyll concentration. We also found that the abundance of starch synthesis-related proteins in *Ca.* L. asiaticus-infected petiole was further increased after the symptom-free prodromal period. Deregulation of starch biosynthesis may be a hallmark physiological change from the asymptomatic to symptomatic stages in HLB-infected trees. Concurrently, our study showed that the salicylic and jasmonic acid content in *Citrus* had a positive correlation with the abundance of phytohormone biosynthesis-related proteins in response to *Ca.* L. asiaticus infection. In addition, an F-type ATPase and an alpha-1,4 glucan phosphorylase presumably were the core nodes in the interactions of differentially accumulated proteins; these results of our study contribute to find out the key *Ca.* L. asiaticus-responsive genes for tolerance and resistance breeding.

## Data Availability Statement

The datasets presented in this study can be found in online repositories. The names of the repository/repositories and accession number(s) can be found below: http://www.proteomexchange.org/, PXD023576. Our data are publicly available under accession number public F16704.

## Author Contributions

DQ and SW conceived and designed the study and revised the manuscript. BL and YZ performed the experiment and wrote the manuscript. SW and FF helped to revise the manuscript. All authors approved the final manuscript.

## Conflict of Interest

The authors declare that the research was conducted in the absence of any commercial or financial relationships that could be construed as a potential conflict of interest.

## Footnotes

1 https://string-db.org/

2 http://www.ncbi.nlm.nih.gov/tools/primer-blast/

## Supplementary Material

Supplementary Figure 1 Quality control validation of MS data.** (A)** Length distribution of identified peptides. **(B)** Distribution of identified proteins in each replicate was shown by a Venn Diagram. **(C)** Volcano Plot representing All identified proteins. Differentially accumulated proteins were shown as red (up-regulation) and blue (down-regulation) spots. **(D)** Peptide Delta Mass Analysis. **(E)** The consistency among three replicates. The X and Y-axis correspond to the number of spectrums for each protein in two replicates, respectively. Spot corresponds to a protein data.

Supplementary Figure 2 PageMan display of results of Wilcoxon rank sum test for significant MapMan functional gene categories that were regulated in leaf petiole of CI1 compared to MI1 and CI2 compared to MI2. Colored boxes represent statistically significant gene groups (*p* value < 0.05). The color scale represents regulation of gene expression. Red, upregulated functional categories; green, downregulated functional categories. The arrows shown MapMan annotation of differentially regulated gene classes.

Supplementary Figure 3 Regulation of carbohydrate metabolic pathway proteins by *Ca.* L. asiaticus infection in leaf petioles. **(A)** Asymptomatic stage (CI1 vs. MI1) and **(B)** symptomatic stage (CI2 vs. MI2). Red squares, significantly up-regulated proteins. Each colored square represents a single annotated gene in a particular pathway.

Supplementary Figure 4 Regulation of proteins involved in ubiquitin-dependent protein degradation by *Ca.* L. asiaticus infection in leaf petioles. **(A)** Asymptomatic stage (CI1 vs. MI1) and **(B)** symptomatic stage (CI2 vs. MI2). Red squares, significantly up-regulated proteins. Each colored square represents a single annotated gene in a particular pathway. There no significantly up-regulated proteins involved in ubiquitin-dependent protein degradation were observed in asymptomatic stage.

Supplementary Figure 5 Differentially accumulated proteins validated using qRT-PCR. **(A)** Results of qRT-PCR analysis of 16 DAPs selected from the comparison *Ca.* L. asiaticus-infected citrus plants with counterpart healthy. Error bars represent the SD of 3 independent experiments. **(B,C)** Correlation between results of relative gene expression from qRT-PCR and relative protein accumulation from proteomics analysis. FC: fold change.

Supplementary Figure 6 Western blotting analysis of psaDb (PS1 reaction center subunit III) and GBSS1 (granule-bound starch synthase 1) accumulation level in leaf petiole at asymptomatic stage (CI1 vs. MI1) and symptomatic stage (CI2 vs. MI2). 20 μg extracted proteins were loaded into each lane, and three replicates were carried out.

Supplementary Table 1 The identification of peptides and proteins based on mass spectrometry proteomics data.

Supplementary Table 2 Differentially accumulated proteins (DAPs) in asymptomatic and symptomatic citrus petiole caused by *Ca.* L. asiaticus infection.

Supplementary Table 3 Gene ontology (GO) and Kyoto Encyclopedia of Genes and Genomes (KEGG) pathway enrichment analysis of differentially accumulated proteins (DAPs) in asymptomatic and symptomatic citrus petiole.

Supplementary Table 4 MapMan annotation of differentially accumulated proteins (DAPs) in asymptomatic and symptomatic citrus petiole.

Supplementary Table 5 Interaction network of differentially accumulated proteins (DAPs) analyzed by ClusterONE embedded in Cytoscape software in asymptomatic and symptomatic citrus petiole.

Supplementary Table 6 Information of primers used in this study.
