# Supplementary figures and images for: Comparative Proteomic Analysis of Sweet Orange Petiole Provides Insights Into the Development of Huanglongbing Symptoms
Source: Front Plant Sci. 2021 Apr 19;12:656997. doi: 10.3389/fpls.2021.656997 (PMC8092123; doi:10.3389/fpls.2021.656997)

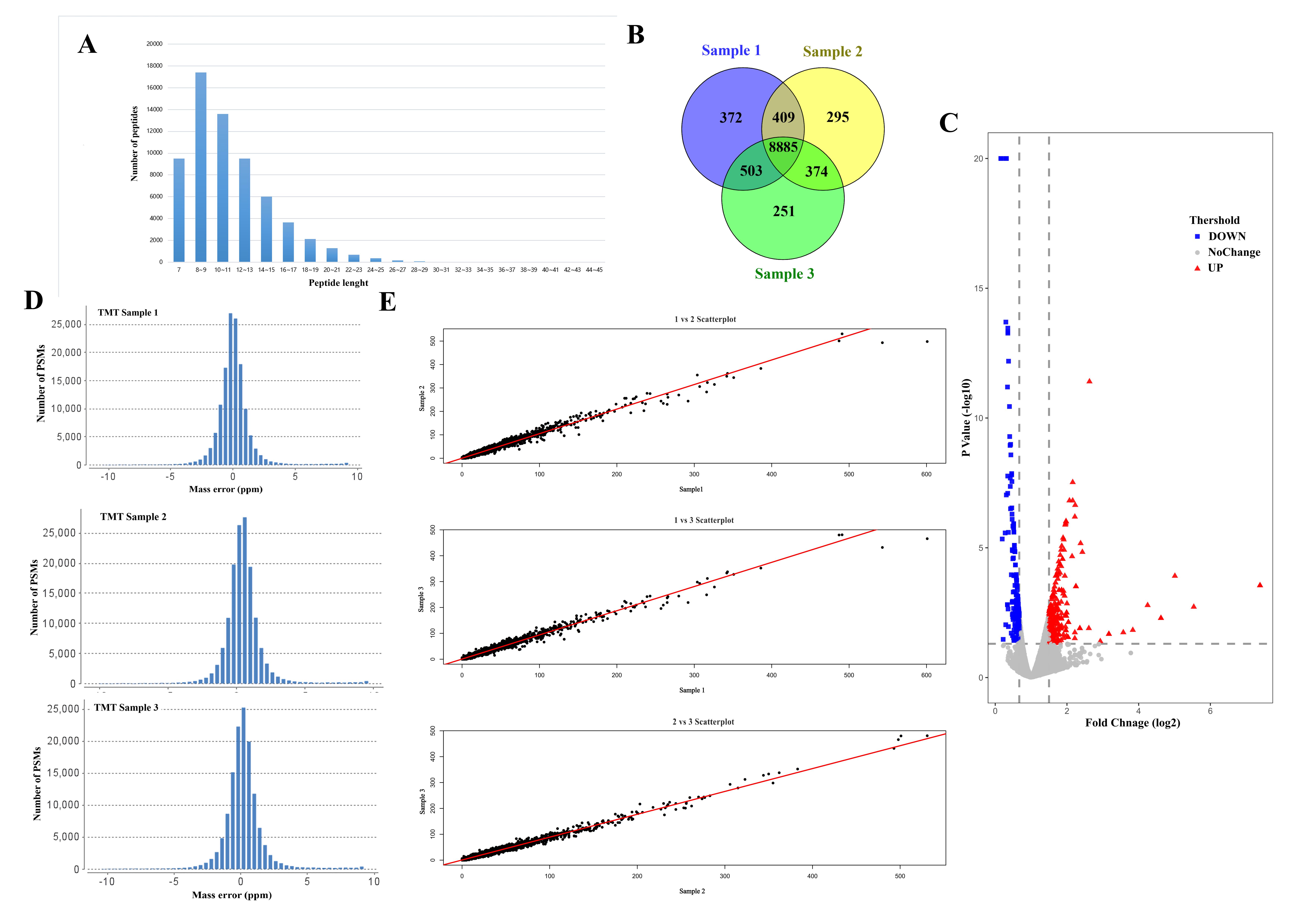

Supplement: Supplementary Figure 1 — Quality control validation of MS data. (A) Length distribution of identified peptides. (B) Distribution of identified proteins in each replicate was shown by a Venn Diagram. (C) Volcano Plot representing All identified proteins. Differentially accumulated proteins were shown as red (up-regulation) and blue (down-regulation) spots. (D) Peptide Delta Mass Analysis. (E) The consistency among three replicates. The X and Y-axis correspond to the number of spectrums for each protein in two replicates, respectively. Spot corresponds to a protein data. [file Image_1.JPEG]

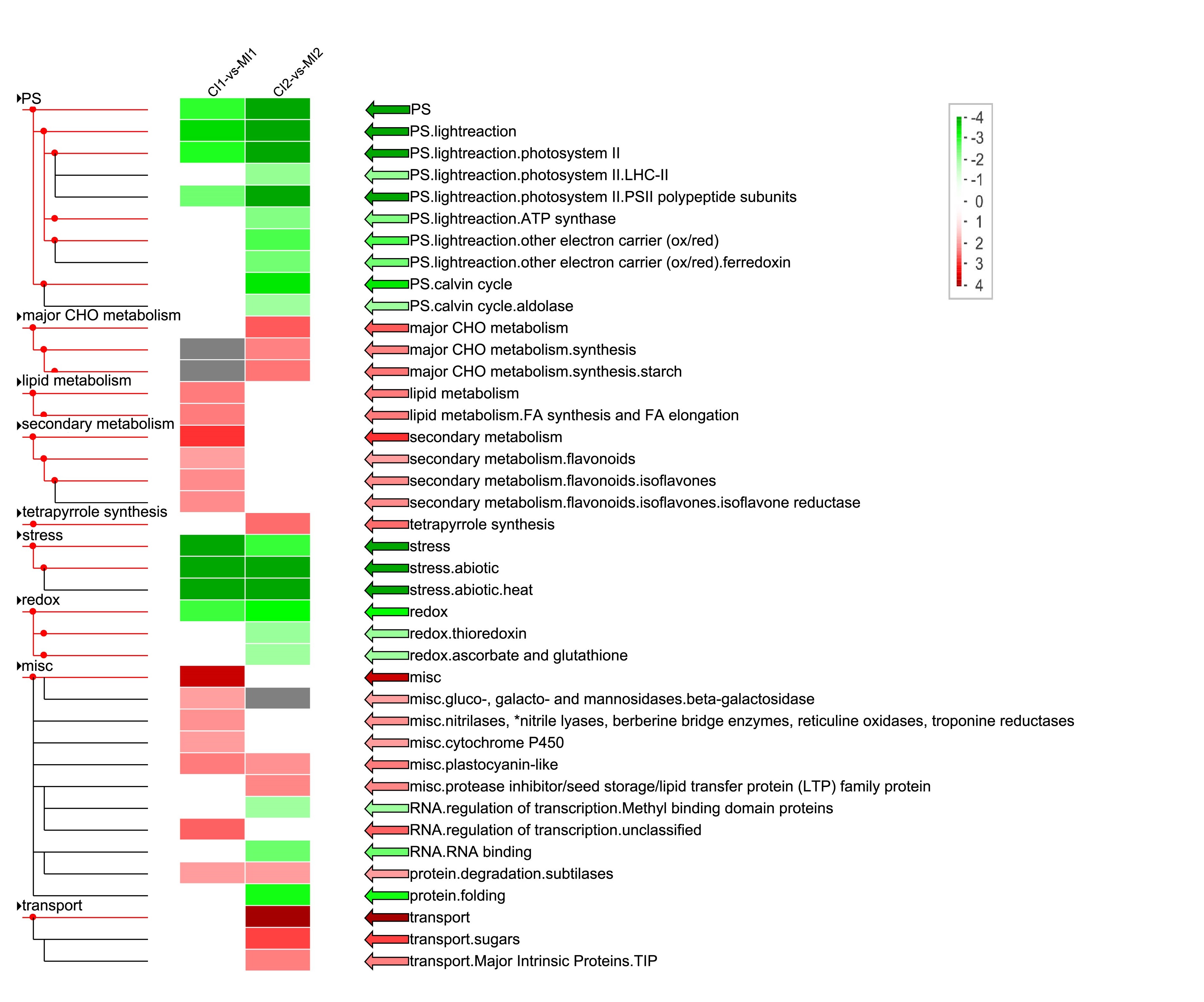

Supplement: Supplementary Figure 2 — PageMan display of results of Wilcoxon rank sum test for significant MapMan functional gene categories that were regulated in leaf petiole of CI1 compared to MI1 and CI2 compared to MI2. Colored boxes represent statistically significant gene groups (p value < 0.05). The color scale represents regulation of gene expression. Red, upregulated functional categories; green, downregulated functional categories. The arrows shown MapMan annotation of differentially regulated gene classes. [file Image_2.JPEG]

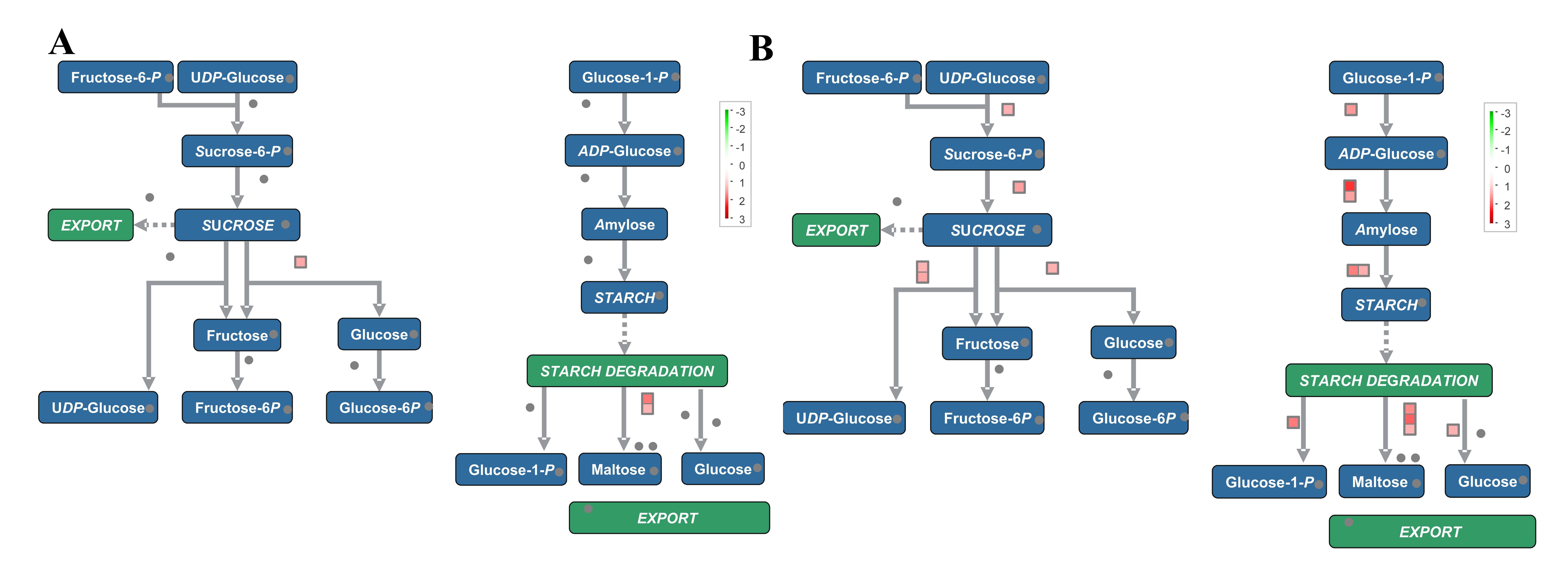

Supplement: Supplementary Figure 3 — Regulation of carbohydrate metabolic pathway proteins by Ca. L. asiaticus infection in leaf petioles. (A) Asymptomatic stage (CI1 vs. MI1) and (B) symptomatic stage (CI2 vs. MI2). Red squares, significantly up-regulated proteins. Each colored square represents a single annotated gene in a particular pathway. [file Image_3.JPEG]

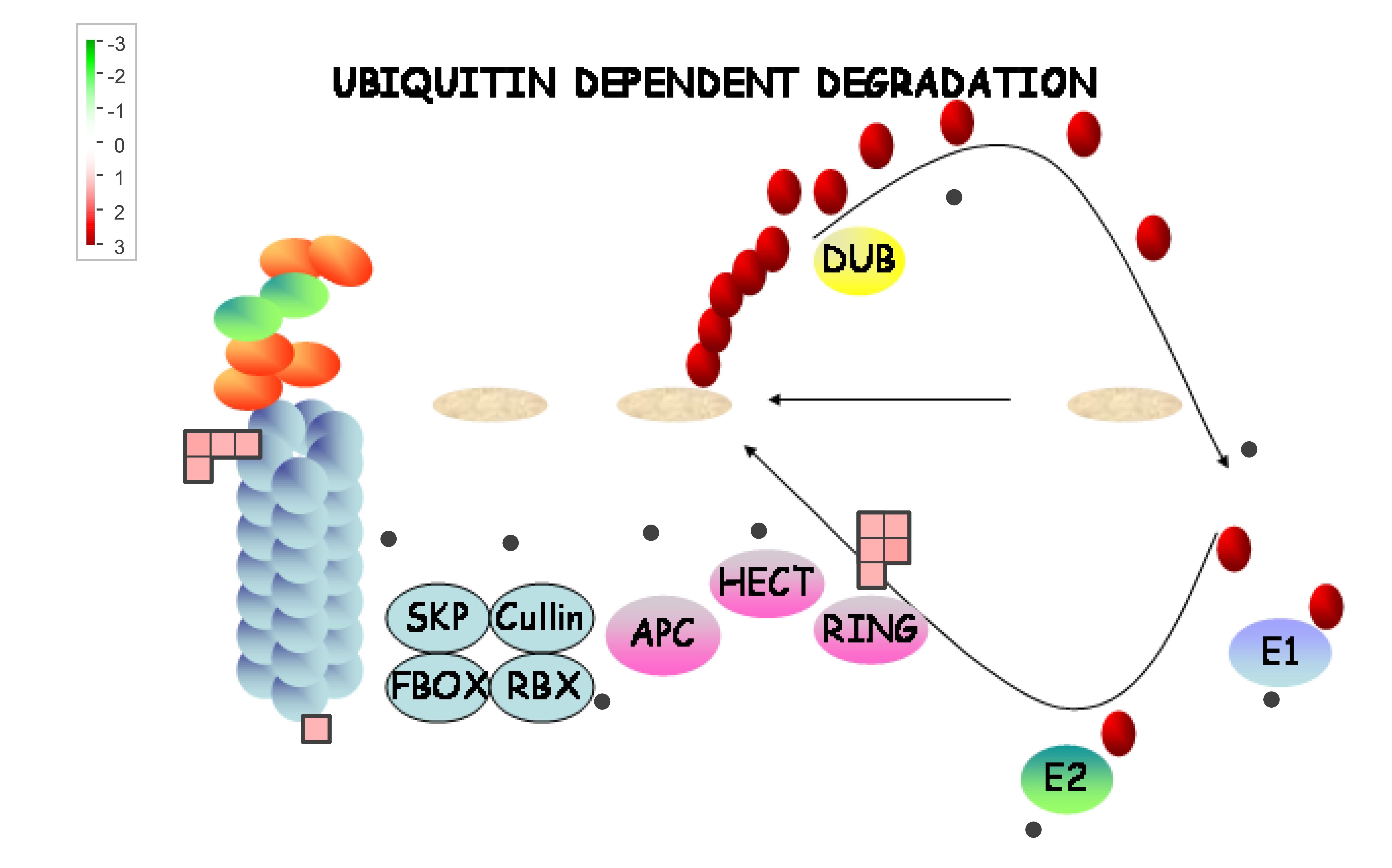

Supplement: Supplementary Figure 4 — Regulation of proteins involved in ubiquitin-dependent protein degradation by Ca. L. asiaticus infection in leaf petioles. (A) Asymptomatic stage (CI1 vs. MI1) and (B) symptomatic stage (CI2 vs. MI2). Red squares, significantly up-regulated proteins. Each colored square represents a single annotated gene in a particular pathway. There no significantly up-regulated proteins involved in ubiquitin-dependent protein degradation were observed in asymptomatic stage. [file Image_4.JPEG]

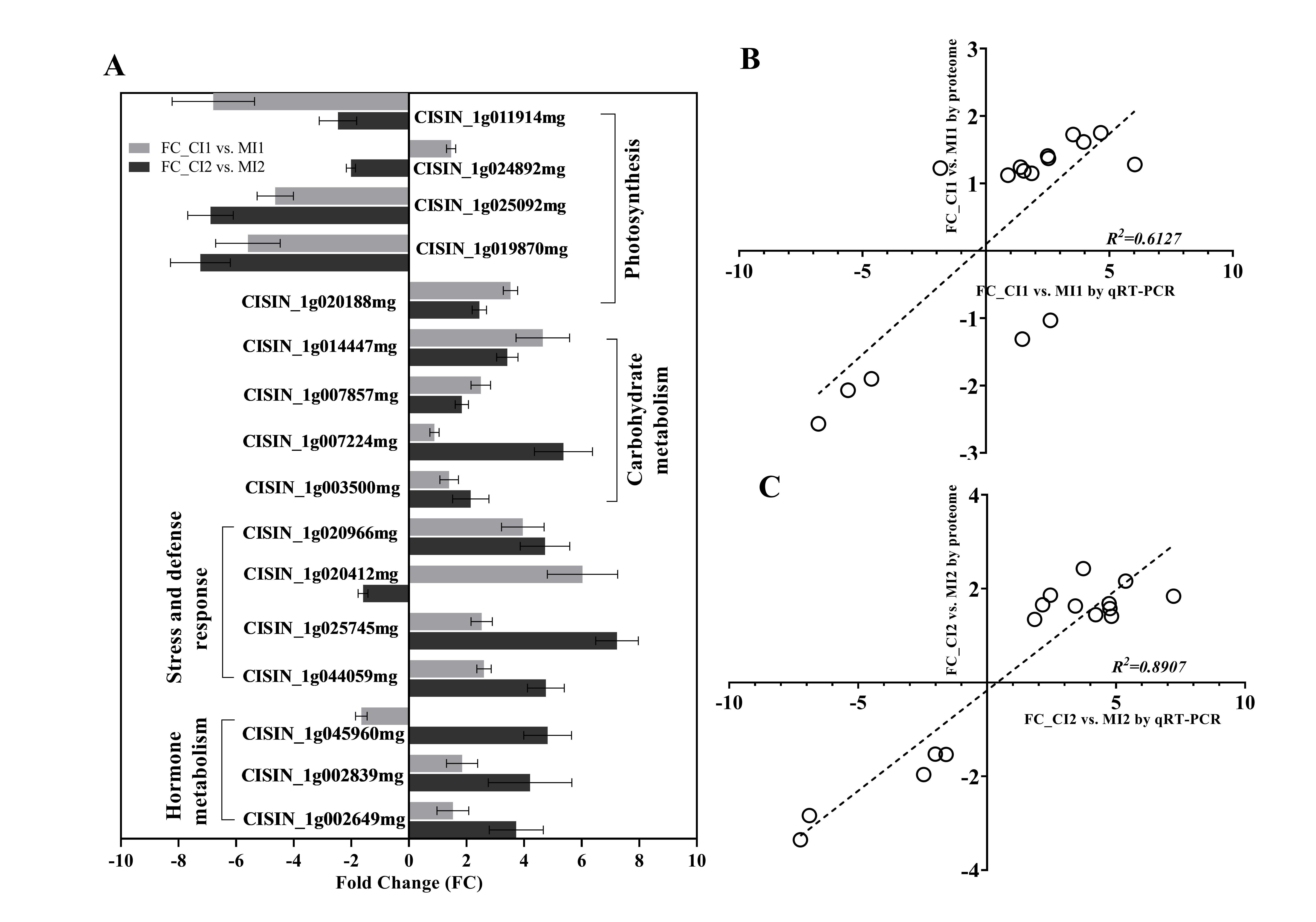

Supplement: Supplementary Figure 5 — Differentially accumulated proteins validated using qRT-PCR. (A) Results of qRT-PCR analysis of 16 DAPs selected from the comparison Ca. L. asiaticus-infected citrus plants with counterpart healthy. Error bars represent the SD of 3 independent experiments. (B,C) Correlation between results of relative gene expression from qRT-PCR and relative protein accumulation from proteomics analysis. FC: fold change. [file Image_5.JPEG]

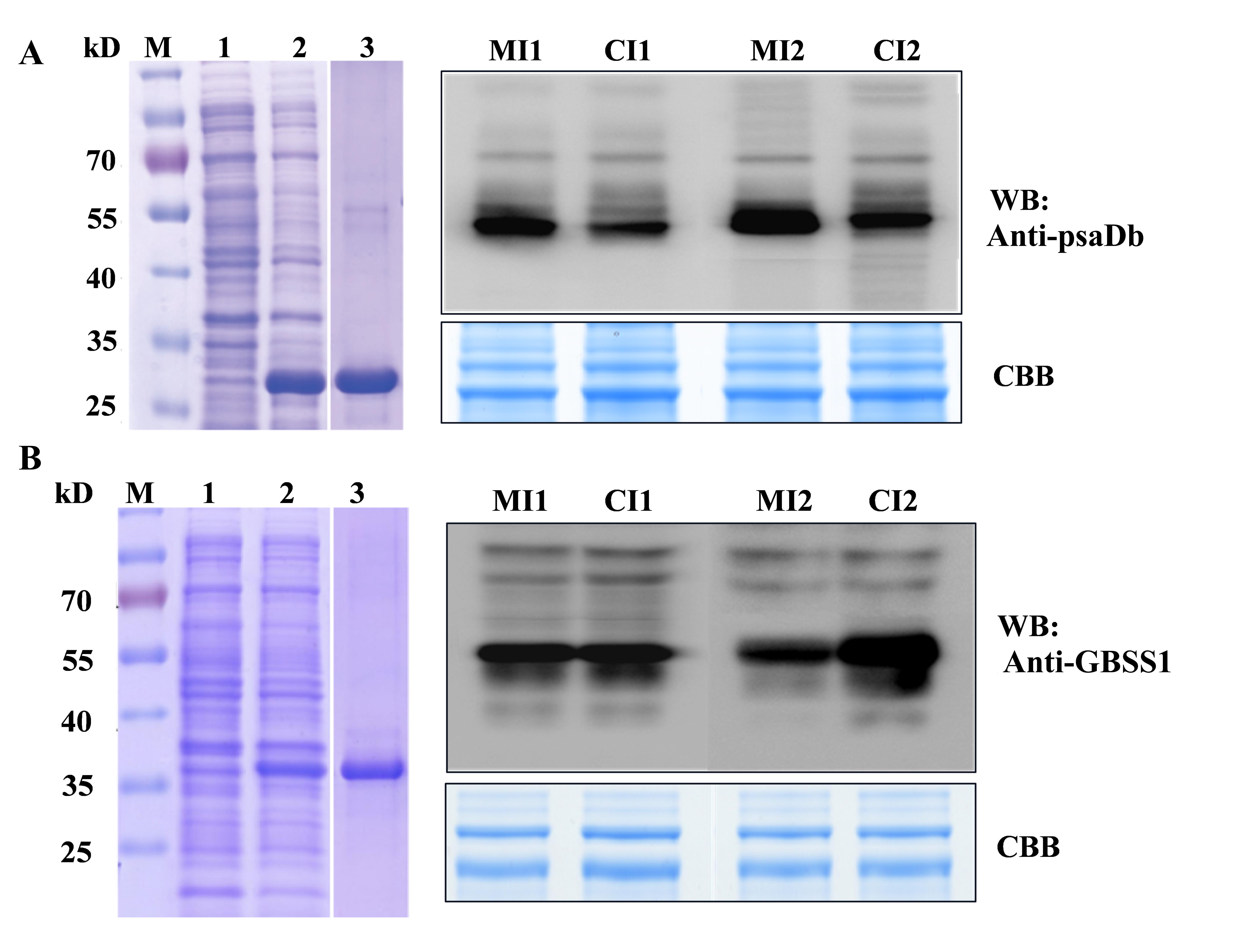

Supplement: Supplementary Figure 6 — Western blotting analysis of psaDb (PS1 reaction center subunit III) and GBSS1 (granule-bound starch synthase 1) accumulation level in leaf petiole at asymptomatic stage (CI1 vs. MI1) and symptomatic stage (CI2 vs. MI2). 20 μg extracted proteins were loaded into each lane, and three replicates were carried out. [file Image_6.JPEG]
