# Supplementary material for: Comparative Proteomic Analysis of Sweet Orange Petiole Provides Insights Into the Development of Huanglongbing Symptoms
Source: Front Plant Sci. 2021 Apr 19;12:656997. doi: 10.3389/fpls.2021.656997 (PMC8092123; doi:10.3389/fpls.2021.656997)
Supplement: Supplementary Table 6 — Information of primers used in this study. [file Table_6.DOCX]

**Table S6. Information of primers used in this study.**

| **Primer name** | **Forward primer sequence(5'-3')** | **Reverse primer sequence(5'-3')** |
| --- | --- | --- |
| **qPCR primers for verifying DAPs results** | | |
| CISIN_1g011914mg | GGGAATGGTGGACACACTCT | TCCAAGCTGTACTGGCGAAG |
| CISIN_1g024892mg | ACCCATTATCCGCCACCATC | GTTGTTCGCTTTCGCCAAGT |
| CISIN_1g019870mg | CCACTTCTGCTCTCGTCGTT | CACTGGTTAGCAGTTCCGGT |
| CISIN_1g025092mg | TCAAGCCCACCCAGATTGTC | ACCAATGAGCACCGTCAGAG |
| CISIN_1g020188mg | TCCGTTGAAGCTAAGCCTGG | CAGTGCCGTGGAAGAACAAA |
| CISIN_1g014447mg | CCACCGTCTCAATCCGTTG | TTCCTCTCTGCAGTTCGGTG |
| CISIN_1g007857mg | CGCGTAGTGAGCAACTCAGA | ATTGGAGGCCATTGTGGAGG |
| CISIN_1g007224mg | CGAGAAAATCCTGCGGTTCG | CAGCTGACTTCGCTCTTTGC |
| CISIN_1g003500mg | TTGTTGCCGGTGCTTTCATC | TCCTCTGGGTCATCCACCAT |
| CISIN_1g020966mg | CCAGCCTGGCAACACTCATA | GGGCAGGTTGTTCTTCTGGT |
| CISIN_1g020412mg | TAACGGCCAGCCAAAAGGAT | GGCCTTGGCCAATAGAGTGT |
| CISIN_1g025745mg | CTCATGCCAAACTGGGGACT | AATGTAAGGCCGACGCTTGT |
| CISIN_1g044059mg | CCTGTGTCGATGAAGCCTGT | CCACCTTGCCTCCTACCTTG |
| CISIN_1g002649mg | AACCCACCATCAAAAGCCGA | CAGCCTCCACCAATATGGCA |
| CISIN_1g045960mg | TCCTTTGTAAGTGAGCGCAA | ACCCTACGAAGAAGGAAGTCT |
| CISIN_1g002839mg | ACATATGGCACTACTCGGCTT | GCGCTAGCTTCCCAATCTTTC |
| **Specific primers for detection of CLas** | | |
| HLB-*16 sRNA* | TCGAGCGCGTATGCAATACG | GCGTTATCCCGTAGAAAAAGGTAG |
